# Imitation of Body Movements Facilitated by Joint Attention through Eye Contact and Pointing in Japanese Monkey

**DOI:** 10.1371/journal.pone.0003704

**Published:** 2008-11-11

**Authors:** Mari Kumashiro, Osamu Yokoyama, Hidetoshi Ishibashi

**Affiliations:** 1 Section of Cognitive Neurobiology, Department of Maxillofacial Biology, Tokyo Medical and Dental University, Tokyo, Japan; 2 Department of Animal Models for Human Disease, National Institute of Neuroscience, Tokyo, Japan; 3 Division of Applied System Neuroscience, Department of Advanced Medical Research Center, Nihon University Graduate School of Medical Science, Tokyo, Japan; 4 Advanced Research Institute for the Sciences and Humanities, Nihon University, Tokyo, Japan; L'université Pierre et Marie Curie, France

## Abstract

Eye contact and pointing are typical gestures in order to direct another individual's attention toward a target. We previously investigated on Japanese monkeys whether joint attention ability encouraged by eye contact and pointing was associated with the imitation of human's actions. The monkeys with the joint attention skills showed the imitation of human's actions. In the current study, we investigated on a monkey whether joint attention ability also facilitated the imitation of human body-movements. Results showed that the monkey being taught eye contact and pointing showed the imitation of human body-movements. These results suggest that the monkeys have basic potential for following another individual's motion, and that what imitation expresses depends on where the monkeys are paying attention. Thus, eye contact and pointing are suitable for directing the monkey's attention toward the human.

## Introduction

The ability of gaze-following has been shown in humans and monkeys [1]–[4]. Joint attention is defined as a process which two individuals attend to a same object while one is following the other's attention [1], [5], [6]. Joint attention thus needs to attend toward the object. In humans, the ability to engage in joint attention is involved in sharing interest in a specific object or event, sharing emotion or communicative intent [5]. Carpenter et al. (1995) reported a positive relation between the joint attention skills and imitative learning in young typically developing children and chimpanzees [7]. Since there had been no studies showing joint attention between humans and monkeys, we initially investigated it [8]. A monkey altered her behavior to a human depending on whether the human's eyes were disguised or not, and pointed at a target at which the human gazed or pointed. From those results, we hypothesized that joint attention ability was pivotal for imitation also in the monkeys, and investigated further on Japanese monkeys whether joint attention ability was associated with the imitation of human's actions [9]. The monkeys who had learned the joint attention skills including eye contact and pointing showed it, meanwhile the monkey who had insufficient eye contact did not but he reproduced the human body-movements. The latter monkey imitated the human's actions only after acquired sufficient joint attention. Thus, results from a series of our studies support our hypothesis [9].

Our proposal for the mechanism that imitation is facilitated by joint attention [9] is described below. If an observer (O) can follow a performer (P)'s gaze direction and an object, O looks not only at the object but also at the P's movements related to the manipulation, and thus O represents the whole action of P and imitates the P's action. If O directs O's attention only to the object, O may represent only some part or the movements of the target. In that case, O may fail to imitate the P's action due to poor representation of the whole manipulation. We, here, define action as behavior including the manipulation of the object, while movement as motion.

Although we previously showed that the monkey reproduced the human body-movements when his head was restrained in order to direct his attention toward it, we did not investigate whether joint attention ability was associated with the imitation of body-movements. In the current study, we investigate it by using the joint attention skills including eye contact and pointing in order to prompt the monkey to direct his attention toward the body parts and movements. Finally, we show another monkey's behavior as the reference of monkeys with mature joint attention ability.

## Results

**Figure 1:**
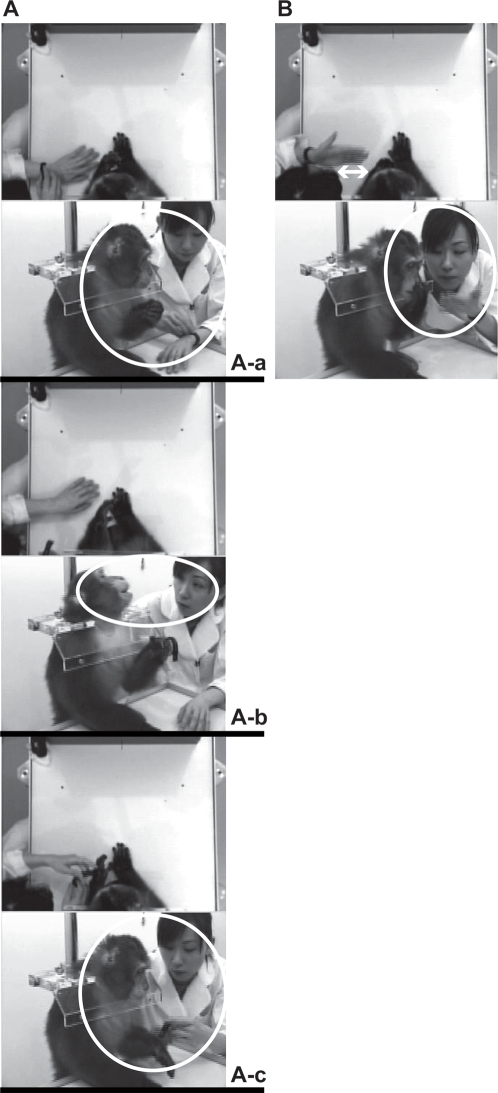
The top and front views during the imitation training. The views showing joint attention during the imitation training (A). The experimenter noticed that the monkey looked at her wrist watch (A-a), and then she also looked at and manipulated it (A-b). Finally, the experimenter put her watch on his arm (A-c). The views showing moving his hand to touch his face (B).

Kin looked between the experimenter's wrist watch and her face alternately during the imitation training, and then the experimenter broke into the training to pay her attention toward her watch with Kin (fig. 1A). The experimenter was able to rather approach to Kin (fig. 1B). There was no object, which was likely to distract joint attention, on the table (fig. 1A, B).

**Figure 2:**
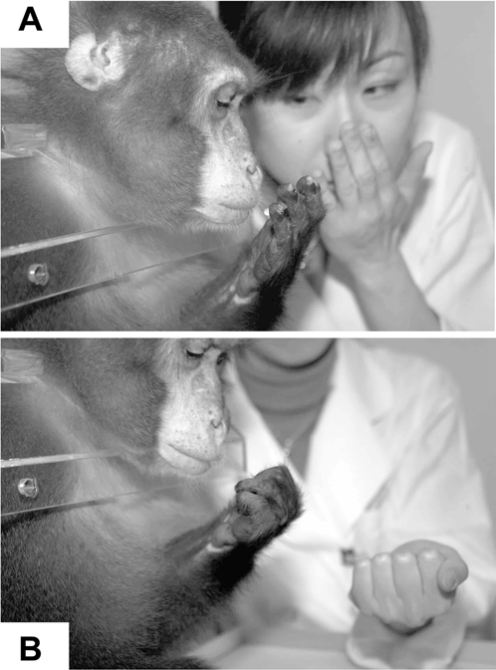
Body-movement imitation. Moving monkey's hand to touch his face (A). Making his fist (B).

**Figure 3:**
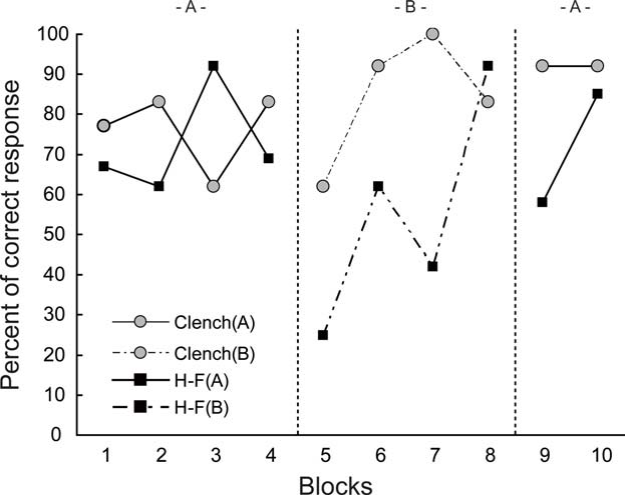
Percentage of correct body-movement imitation. Performance was different depending on the type of models, especially decreased in imitating the model related to the face. ‘-A-’ and ‘-B-’ stand for each of two experimenters.

The imitation of body-movements: Kin monitored the human's body-movements and in turn the Kin's hand and his body-movements for a few seconds each time. Kin imitated, touching his own face (H-F) and clenching his hand into a fist (Clench), with his left hand (fig. 2). There was no difference in the Kin's response between experimenter A and B (p = 0.655, Wilcoxon rank sum test). In the test phase of the experimenter A, an average percent of four blocks in Clench and H-F was 76% and 72% respectively. When the experimenter B changed off the experimenter A, a percent of the first block in Clench and H-F was 62% and 25% (fig. 3) respectively. Percentages increased to the level of experimenter A condition. After another change, a percent of the first block in Clench and H-F was 92% and 58% respectively. Percentage for the H-F model increased in the next block. Thus, percentage in H-F but not in Clench dropped when the experimenter changed.

### Follow-up: natural imitation

**Figure 4:**
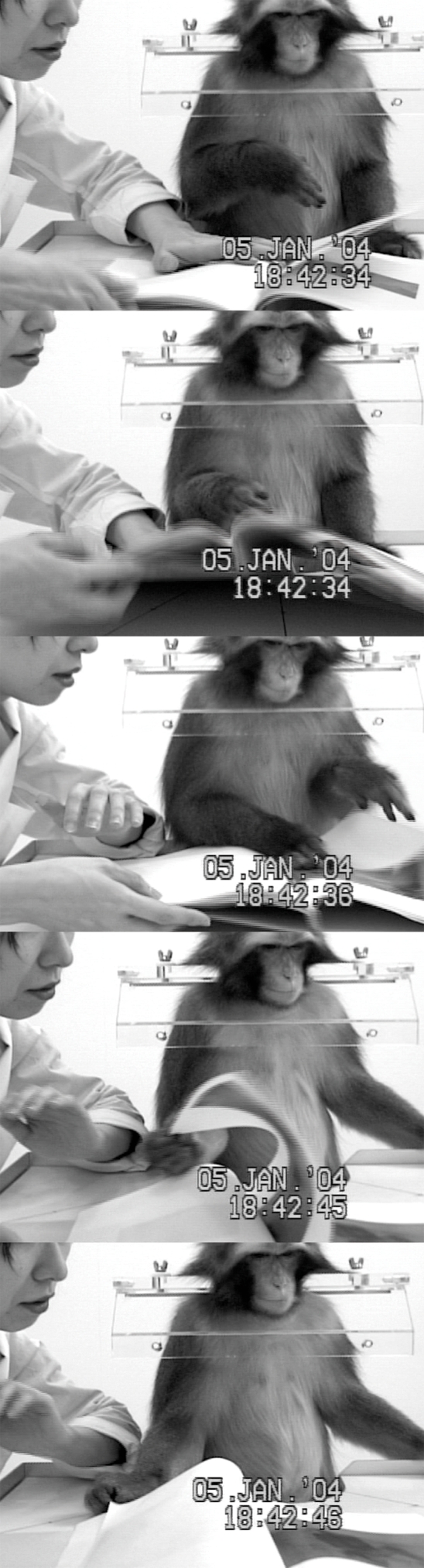
Sequential photographs of turning the pages. The monkey watched the experimenter turning pages and turned the page (top three panels). The monkey turned another page (bottom two panels).

At the beginning in the book session, Pin tried to bite or fiddle around the book just after the experimenter presented it to Pin. Immediately, the book was taken away from Pin. After the experimenter pointed at the corner of the pages and carefully turned page by page, Pin also imitated it (fig. 4). Since then, this action was included in the repertoire of Pin's behavior and she became to turn the page voluntarily.

## Discussion

We conducted for one monkey the imitation training through the joint attention skills and showed that he imitated the human body-movements. Our procedure was effective in directing the monkey's attention toward the human models for cross-species imitation. The current and previous data suggest that there are three important matters for imitation facilitated by joint attention between the humans and monkeys [9].

First, the imitation training requires two steps, eye contact and communicative pointing [9]. Second, confusing movements to distract monkey's attention during the imitation training should be eliminated by the experimenter as far as possible, except for joint attention situation (fig. 1A). It is very important to discriminate the monkey's looking at the confusing movements from the monkey's looking at the available object. The former is passive, while the latter is active. That difference might profoundly influence on imitation. In the former, the repetition of unnecessary movements may be effective over time and result in an inappropriate imitation, since the experimenters seldom notice that they conveyed misleading information to the monkey. In the latter, as was the case in the current study, the experimenter was able to pay her attention to the same object jointly with the monkey, and it might help to shorten the distance between the experimenter and monkey (fig. 1B). Third, we should keep in mind that it took a bit of time for the monkey to adapt to imitation related to the face (fig. 3). Gaze direction is an important cue in the perceptual processing of facial display of emotion [10]. We never stared at the monkey's eyes when not necessary during the training. Although the monkey sometimes watched his own hand or its movements (fig. 2), it would appear that the monkey represented and rehearsed the body-movements of imitation.

Our previous study has shown that the monkey reproduced the human body-movements with the head positioner even though the joint attention ability was incomplete [9]. In this study, the imitation of human body-movements was observed for the monkey without the head positioner but with the joint attention gestures. As we proposed previously, although monkeys have basically the potential for following another individual's motion, what imitation expresses depends on whether it mediates communicative circuit or not. The imitation of another individual's action requires observer's attention toward a sequence of body-movements, while an initial reproduction of movements may derive from one's attention to a local motion. It is innately present based on the visuo-motor function circuit. Human's neonates are able to reproduce simple facial movements, such as a tongue protrusion and mouth opening [11], [12]. In chimpanzees, at less than seven days of age the chimpanzees could imitate facial gestures [13]. Infant macaques also reproduced oral models of humans although its time window was narrower than in chimpanzees [14]. Jacobson (1979) found a link between the sight of a moving pen and tongue protruding in 6-week-old infants [15]. Thus, the moving stimulus is enough for triggering the body-movements. This elemental function is probably beneficial in following the mother, or in acting in the external world including the communicative partner. Natural imitation shown by our studies seems to be the behavior modulated from this elemental function via the joint attention function since the monkey with insufficient eye contact was not able to imitate the human's action [9]. Joint attention ability may result in a fundamental set of cognitive ability which affects the development of imitation or other social behavior. Further studies are necessary to discern which brain mechanisms are responsible for imitation.

Some researchers propose that the basis of action understanding [16], involved in communicative function [17], is related to mirror neurons in monkey ventral premotor cortex (F5) that are activated during both own movements and observations of familiar actions performed by another individual [18]–[21]. Oztop et al (2006) mentioned on the basis of our previous study that mirror neuron system, related to action understanding [18] or imitation, must be augmented by the attention system [22]. If the monkey acquires faithful imitation based on the joint attention, the mirror neurons might not be sensitive to this type of imitation due to the absence of a particular purpose. It may not respond until the new action acquired by natural imitation is included in the repertoire of aimed behavior.

The implication of our studies contributes significantly to the study of imitation and monkeys. The series of our studies is one of the first to begin to associate imitation ability with joint attention in monkeys. This was done by the imitation training after teaching the joint attention skills. Furthermore, joint attention of the imitation training was valid for shortening the distance between the human and monkey. Although not conclusive, this supports our hypothesis that joint attention ability plays an important part in raising the monkey's interests in the human. Accordingly, we conclude that joint attention gestures facilitate monkey's imitation of human models.

## Materials and Methods

### Subjects

The subjects were two Japanese monkeys, *Macaca fuscata* (Kin: male, 11 kg, Pin: female, 7 kg). Each subject was housed in a single cage. The subjects could drink water freely in the cage. Monkey chow was given 100∼200 g daily. Small amount of fresh apple and sweet potato was given once a day in the cage. Kin was trained in an isolation box (150 cm wide, 150 cm depth, and 185 cm high), seated comfortably in a primate chair (Muromachi: Kikai, Co., Ltd), facing an experimenter at a distance of 60–90 cm. Two experimenters were involved in a training or test. Experimenter A was a main trainer. Experimenter B took out Kin from the cage and became familiar with Kin. This study was approved by the Animal Care and Use Committee of the Tokyo Medical and Dental University, and all husbandry and experimental procedures were in accordance with the guide for the Care and Use of Laboratory Animals of the National Research Council (1996) and the Guidelines for Animal Experiment at Tokyo Medical and Dental University.

### Training

**Figure 5:**
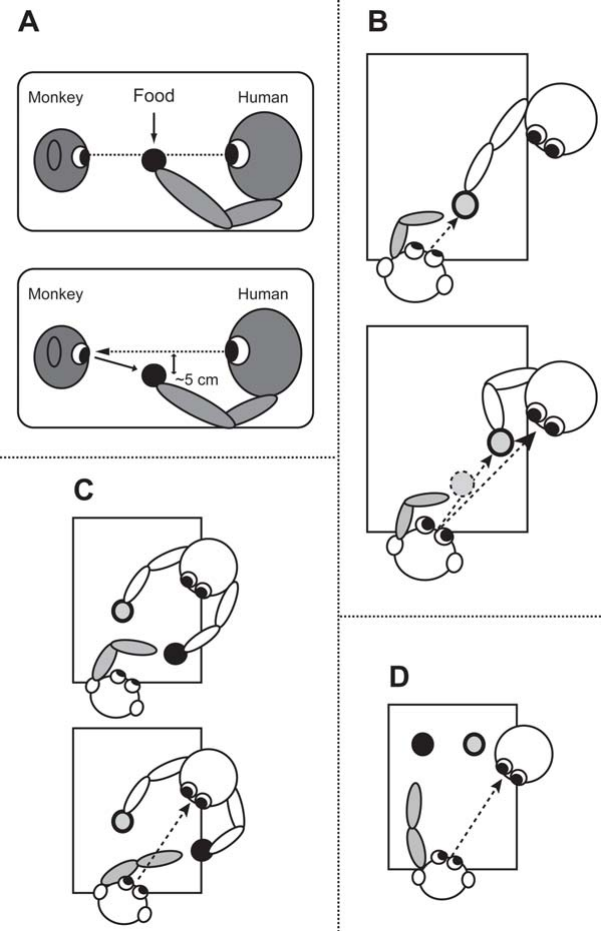
Schematic illustration of the eye contact and pointing training. For the eye contact training, the experimenter firstly presents a piece of food in front of monkey's eyes. Next, the experimenter gradually lowers the position of the food (A). The experimenter moves the food back and forth in order to direct the monkey's attention toward her. The experimenter gives the monkey the food when she realized that the monkey looked at her (B). For the pointing training, the experimenter firstly presents the food between the experimenter and the monkey at the chest level. Then, the experimenter pulls nearer her hand holding the food, when the monkey begins to reach for the food (C). The experimenter gives the monkey the food when the monkey repeatedly reaches for the food (D).

First step was eye contact. The experimenter prepared food, such as sweet potatoes or apples, for which the subject showed the greatest preference. The experimenter cut the food into small slices, within two centimeters length, two centimeters width and about five millimeters thick. Food was placed in a transparent or opaque acrylic container. The experimenter sat as having the container diagonally to the front of the subject. The experimenter gave a piece of food to the subject for several times. After the acclimatization, the experimenter showed the subject the food at the eye level (see, Kumashiro et al., 2002 and fig. 5A, 5B). Then, the experimenter quickly moved the food position downward about five centimeters and then moved it back in place or sometimes from side to side while the experimenter was gazing at the monkey's eyes. By the three or more repetitions of that, the subject voluntarily came to look at the experimenter's eyes. Next, the experimenter moved the food back and forth. As soon as the experimenter met successfully the subject's gaze, she gave the subject the food. The experimenter sometimes changed own position and the position of the food here and there to prevent the subject from associating food with a particular location. The experimenter checked whether the subject did not gaze at the experimenter or not when she looked at the subject's eyes without the food container or objects in which the subject was interested. If the subject occasionally averted the experimenter's gaze in that context, it was judged that the looking at the experimenter's eyes was not the response by association between eyes and rewards. On the contrary, if the subject had gazed at the experimenter's eyes regardless of contexts or where the experimenter's face used to be located, we would have re-trained eye contact from the beginning. Fortunately, it was not observed in the current study. To make enough eye contact, this training required for two days.

Second step was communicative pointing. After the establishment of eye contact, the experimenter began the pointing training. The experimenter showed the subject two pieces of food as having them with both hands (see, Kumashiro et al., 2002 and fig 5C). According to Vygotsky (1978), the developmental origins of pointing are the infant's unsuccessful reaching for objects to which the mother responds by giving him the object [23]. Then, we reproduced the situation in our experimental room. The experimenter held the food at monkey's fingertips and pulled the experimenter's hand when the monkey seized the food (fig. 5B). The subject, after the repetition, began to extend its arm toward the food while the subject was looking at the experimenter. When the subject chose between the two and reached for the one, the experimenter immediately gave it the food. When the subject became to extend its arm toward the food, the experimenter gave it the food only after eye contact. After that, the experimenter moved the food container at various locations, and then the experimenter pointed at the food into the container. When the subject alternately looked at the food and the experimenter, the subject obtained the food. The subject used the preferred hand for pointing. The distance between the subject's fingertip and the food location was gradually increased up to about 70 cm (fig. 5D). Then, the subject pointed at the distant or various food positions. The subject was trained for six days. The experimenter always communicated with the subject by using eye contact and pointing.

We trained the subject to imitate the two body-movements for nine days after the eye contact and pointing training (see, Kumashiro et al., 2003). The experimenter cut an apple or sweet potato in a small piece to allow the subject to consume easily and to prepare for the next trial soon. We uniquely designed a simultaneous-multi training. We conducted two imitation interventions within one session. Since the experimenter presented the subject with arbitrarily-assigned models, the variant movements of body parts could be distinctly visible and come to attract the subject's attention. At first, the experimenter faced to the subject and moved the experimenter's hand to touch the face (H-F) repeatedly. If the subject's hand was moved near the face, the experimenter immediately gave a piece of food to the subject. After monkey's imitation of this model, the experimenter showed the Clench model while moving five fingers to make a horizontal fist with the elbow on the table or raised the clenched hand and swung it downward. The experimenter alternated the H-F and Clench model throughout the session. If the subject began to imitate, to clarify each imitation, the experimenter placed her hand palm down on the table before the presentation of each model. If the subject also started imitating it, the experimenter presented each model after she put her hand on the table. The experimenter generally entered the experimental room with nothing except for the food container because we needed to eliminate extra movements of eye or hand in the experimenter. In the H-F model, the experimenter looked at the subject's hand and face being touched but not his eyes. If the subject did not touch to the face or clench, the experimenter looked and pointed at the appropriate body parts. Thus, we instructed imitation by the experimenter's pointing at the subject's body parts instead of shaping or molding of subject's hand.

### Test

After nine days of training, the degree of completion in the imitation of body-movements was tested by two experimenters, experimenter A and B. All 250 trials were divided into ten blocks of 25 trials. The criterion of correct response in Clench model was that the subject formed the clenched fist and maintained the hand shape of the model over one second. The criterion of correct response in the hand-to-face model was that the subject touched the highest part in the face with his hand. Each trial was started after the experimenter and subject placed their hands on the table.

### Follow-up: imitation of a human's action

We observed Pin's behavior after about four years from when she started to use eye contact and communicative pointing. This monkey was previously tested for the bidirectional-communication between the monkey and human [8], and showed natural imitation [9]. As the follow-up, the experimenter presented a book in front of Pin. After that, the experimenter turned a few pages of the book in Pin's view. Then, the experimenter presented the book to Pin and pointed at the corner of the page.
